# Navigation by modified and dynamic intraoperative cholangiography during laparoscopic subtotal cholecystectomy for difficult gallbladder

**DOI:** 10.1016/j.radcr.2023.01.026

**Published:** 2023-02-11

**Authors:** Fumio Chikamori, Ryo Yamada, Koji Ueta, Kazuhisa Onishi, Mitsuteru Yoshida, Nobuyuki Tanida, Hiromichi Yamai, Hisashi Matsuoka, Norihiro Hokimoto, Sunao Uemura, Jun Iwabu, Kai Mizobuchi, Akira Marui, Niranjan Sharma

**Affiliations:** a Department of Surgery, Japanese Red Cross Kochi Hospital, 1-4-63-11 Hadaminamimachi, Kochi, 780-8562 Japan; b Adv Train Gastroint & Organ Transp Surgery, 12 Scotland St, Dunedin, 9016, New Zealand

**Keywords:** Acute cholecystitis, Pericholecystic abscess, Percutaneous transhepatic gallbladder drainage, Laparoscopic subtotal cholecystectomy, Intraoperative cholangiography, Difficult gallbladder

## Abstract

We used modified and dynamic intraoperative cholangiography (IOC) navigation during laparoscopic subtotal cholecystectomy for difficult gallbladders. We have defined an IOC that does not open the cystic duct as a modified IOC. Modified IOC methods include the percutaneous transhepatic gallbladder drainage (PTGBD) tube method, the infundibulum puncture method, and the infundibulum cannulation method. Case 1 was chronic cholecystitis after PTGBD for acute cholecystitis with pericholecystic abscess. In this case, modified IOC was performed via PTGBD, and biliary anatomy and incarcerated stone were confirmed. Case 2 was chronic cholecystitis after endoscopic sphincterotomy for cholecystocholedocholithiasis. In this case, modified IOC was performed via gallbladder puncture needle, and biliary anatomy and incision line were confirmed. The target point on the laparoscopic image was determined by moving the tip of the grasping forceps under modified IOC, which we call modified and dynamic IOC. We conclude that the navigation by the modified and dynamic IOC via PTGBD tube or puncture needle is useful to identify biliary anatomy, incarcerated gallbladder stone, and safe incision line during laparoscopic subtotal cholecystectomy .

## Introduction

We have reported early scheduled laparoscopic cholecystectomy following percutaneous transhepatic gallbladder drainage (PTGBD) for acute cholecystitis in elderly and complicated patients [1]. The dissection along with the inner layer of the subserosal layer of the gallbladder [2] is easy in early surgery, however, it becomes difficult because of fibrosis in delayed surgery. Surgeons cannot control the duration between the onset and the patient's arrival. Chronic cholecystitis after PTGBD is one of the risk factors for a difficult gallbladder [3]. Sticking to total cholecystectomy for difficult gallbladders leads to risks such as damage to the liver bed, increased bleeding, and bile duct injury. Laparoscopic subtotal cholecystectomy (LSC) is an effective surgical technique to avoid biliary and vascular injuries when one cannot safely dissect and identify the structures in the Calot's triangle [4]. We have reported the usefulness of modified and dynamic intraoperative cholangiography (IOC) during laparoscopic cholecystectomy to avoid biliary injury [1,5]. Here we report the navigation by modified and dynamic IOC via PTGBD tube or puncture needle that can identify biliary anatomy, incarcerated gallbladder stone, and safe incision line during LSC.

## Case 1

A 64-year-old female was referred to our department with complaints of fever (37.5°C) and back pain. Past medical history showed acute cholecystitis 3 months ago which was conservatively treated with an antibiotic. On admission, the patient had a heart rate of 68/min, a blood pressure of 134/88 mmHg, and a respiratory rate of 13/min. Her body temperature was 36.2°C. Her height was 154 cm, body weight was 63 kg, and body mass index was 26.6 kg/m^2^. She complained of back pain and tenderness in the right hypochondrium.

Laboratory results showed hemoglobin 12.1 g/dL (normal range, 13.5-17.4), white blood cell count 5560/μL (3500-8000), platelet count 29.7 × 10^4^/μL (12.3-33.1), total bilirubin 0.5 mg/dL (0.3-1.3), alanine transaminase 52 U/L (5-27), aspartate aminotransferase 44 U/L (10-32), lactate dehydrogenase 197U/L (106-211), alkaline phosphatase 243U/L (109-344), γ-glutamyl transpeptidase 173 U/L (8-45), blood urea nitrogen 10.7 mg/dL (8-20), creatinine 0.66 mg/dL (0.36–1.06), prothrombin time (PT) 11.6 seconds (9-12), PT% 109% (70-130), international normalized ratio 1.0, activated partial thromboplastin time 27.1 seconds (20-35) and C-reactive protein 8.91 mg/dL (<0.16). These results indicated that the patient had recurrent acute cholecystitis.

**Fig. 1:**
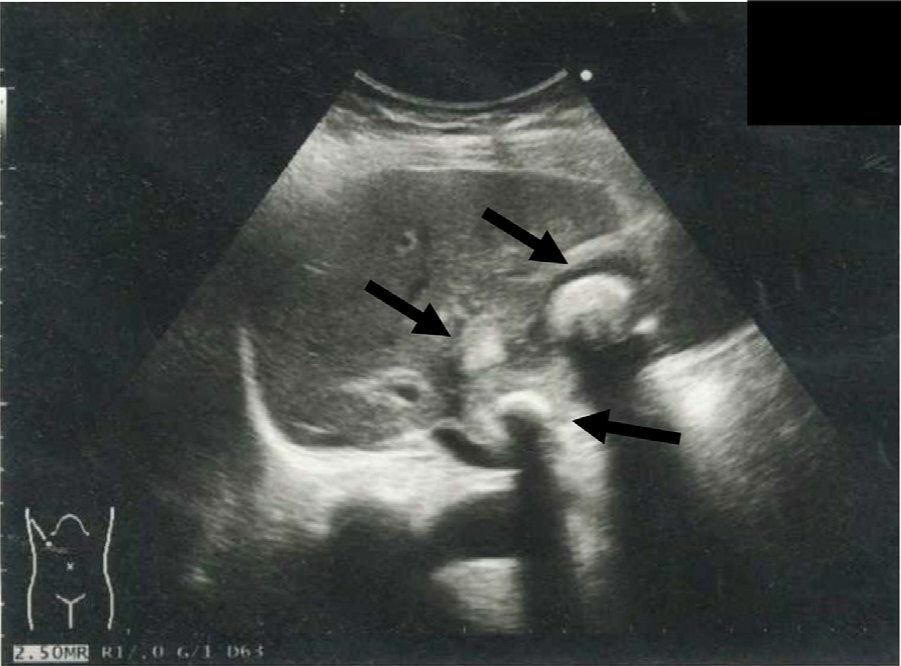
Abdominal ultrasonography before PTGBD shows high echogenic multiple stones (arrow) in the gallbladder. PTGBD, percutaneous transhepatic gallbladder drainage.

**Fig. 2:**
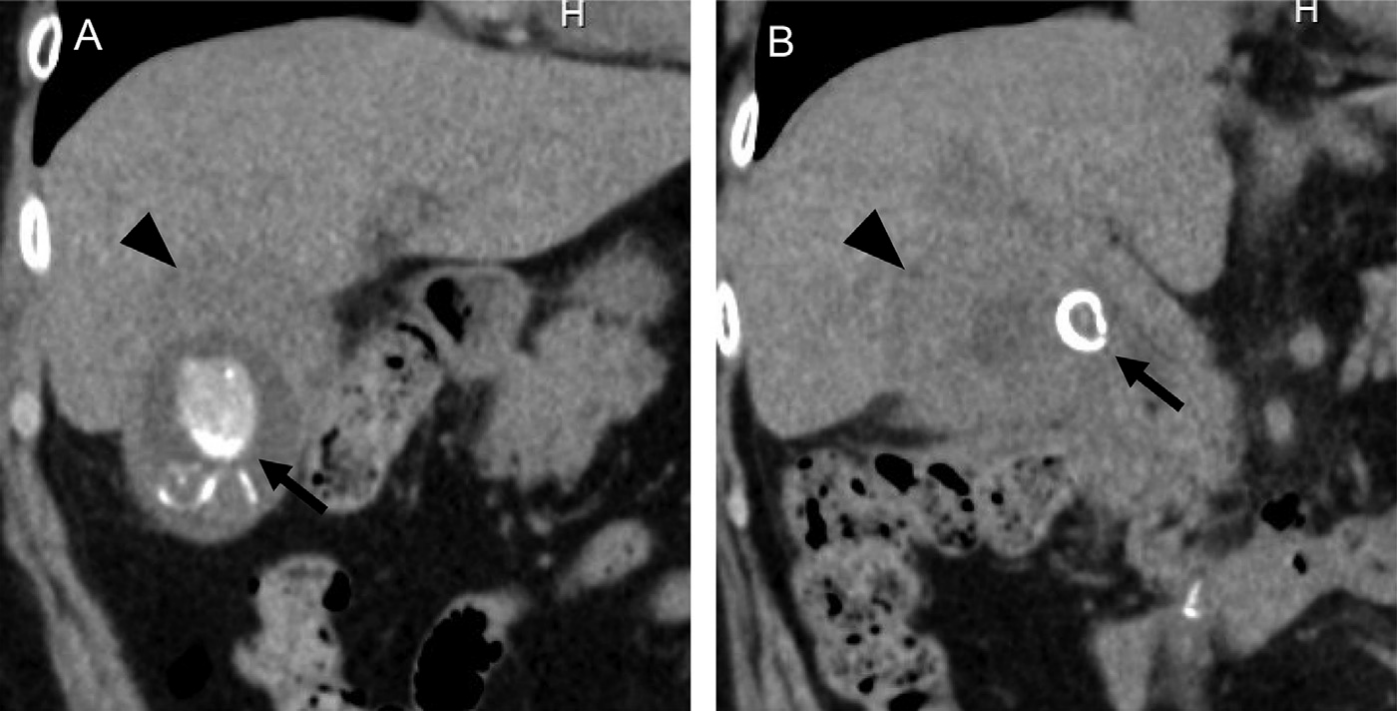
(A) Coronal image of abdominal plain CT shows multiple gallbladder stones (arrow) and wall thickening (arrowhead). (B) Another coronal image of abdominal plain CT shows a stone just right outside the bile duct (arrow). CT, computed tomography.

**Fig. 3:**
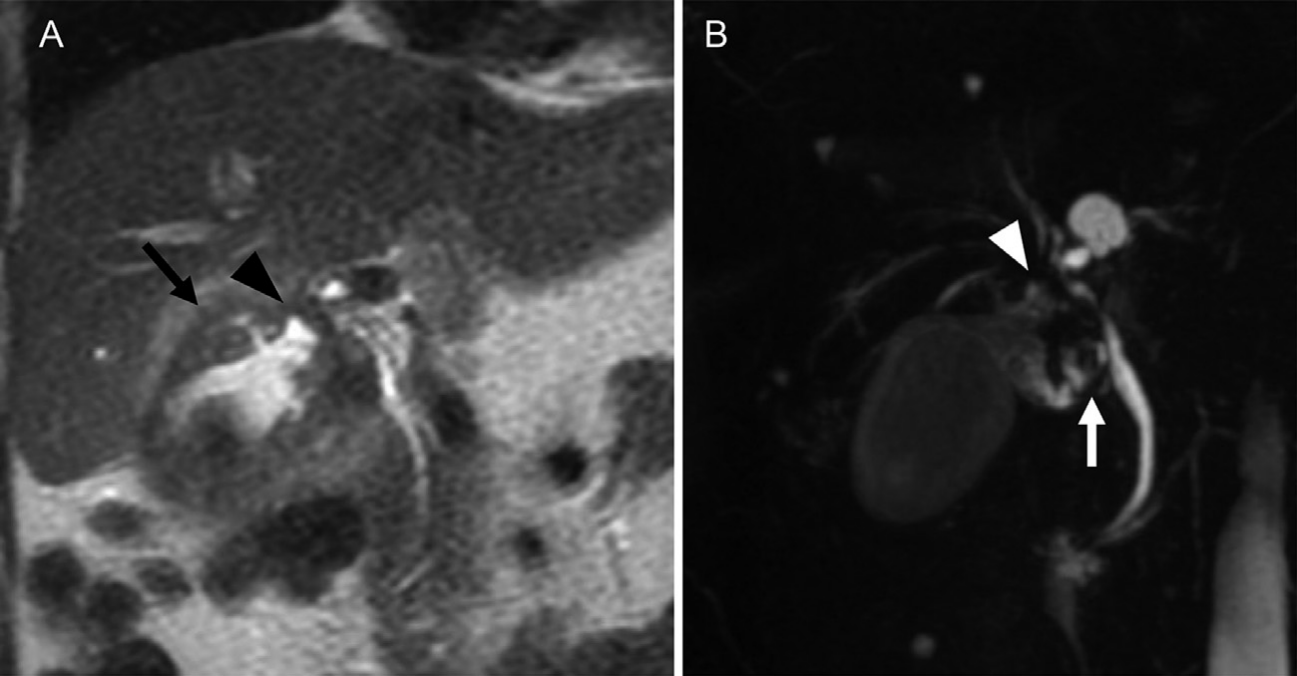
(A) Coronal image of T2-weighted MRI shows pericholecystic fluid collection (arrowhead) and wall thickening of the gallbladder (arrow). (B) MRCP shows incarcerated stone (white arrow) and pericholecystic fluid collection (white arrowhead). MRI, magnetic resonance imaging; MRCP, magnetic resonance cholangiopancreatography.

**Fig. 4:**
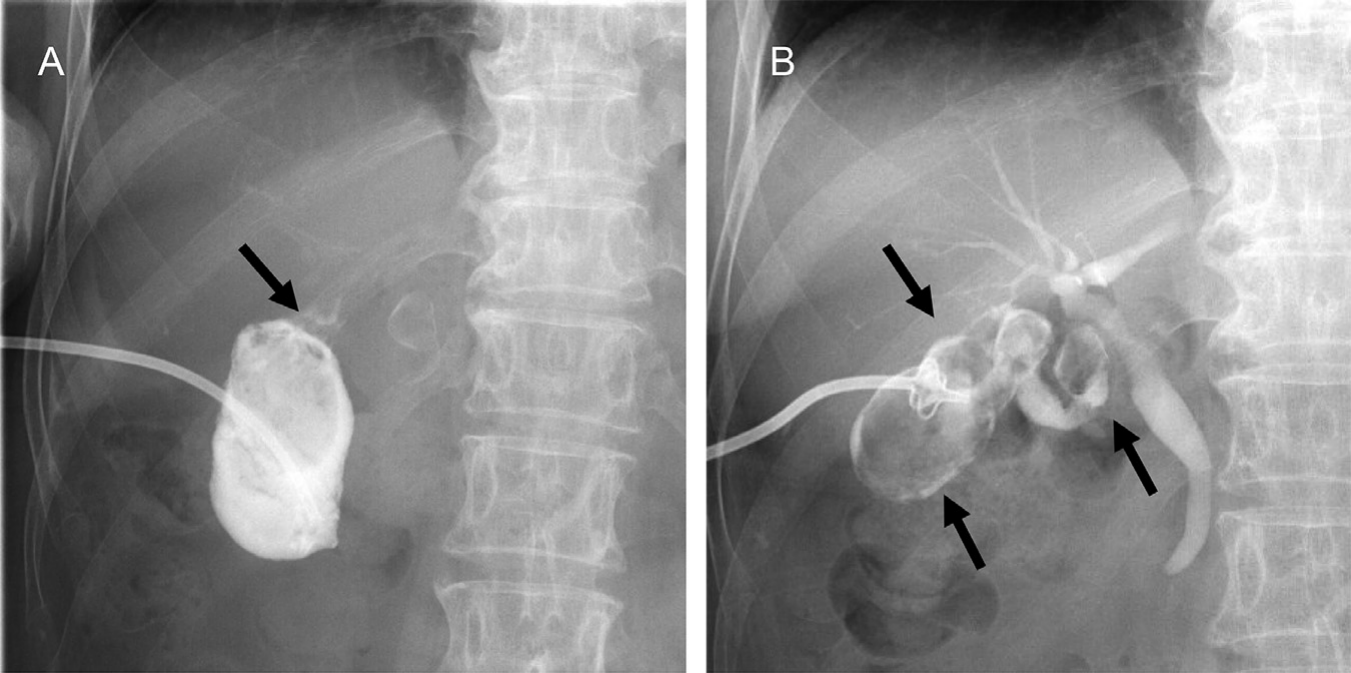
(A) Cholecystography via PTGBD tube shows perforation site (arrow). (B) Cholangiography 2 months after PTGBD shows multiple gallbladder stones (arrow) without pericholecystic abscess. PTGBD, percutaneous transhepatic gallbladder drainage

Abdominal ultrasonography revealed multiple stones in the gallbladder (Fig. 1). On plain abdominal computed tomography (CT), swelling and wall thickening of the gallbladder were detected (Figs. 2A and B). Abdominal magnetic resonance imaging (MRI) and magnetic resonance cholangiopancreatography also showed multiple gallbladder stones and an impacted gallbladder stone in the infundibulum-cystic duct junction. A small amount of fluid around the gallbladder neck was present (Figs. 3A and B). A diagnosis of recurrent acute cholecystitis with pericholecystic abscess was made. She was treated with intravenous antibiotics, and PTGBD was attempted. An 8 Fr. drainage tube was inserted into the gallbladder and 50 mL of white-yellow pus was aspirated. The cholecystography revealed perforation of the gallbladder (Fig. 4A). The culture of the pus aspirated from the gallbladder returned *streptococcus anginosus*. Following PTGBD, her condition improved, and she was discharged 6 days after PTGBD. Interval surgery was planned 3 months after PTGBD when the pericholecystic abscess resolved (Fig. 4B).

**Fig. 5:**
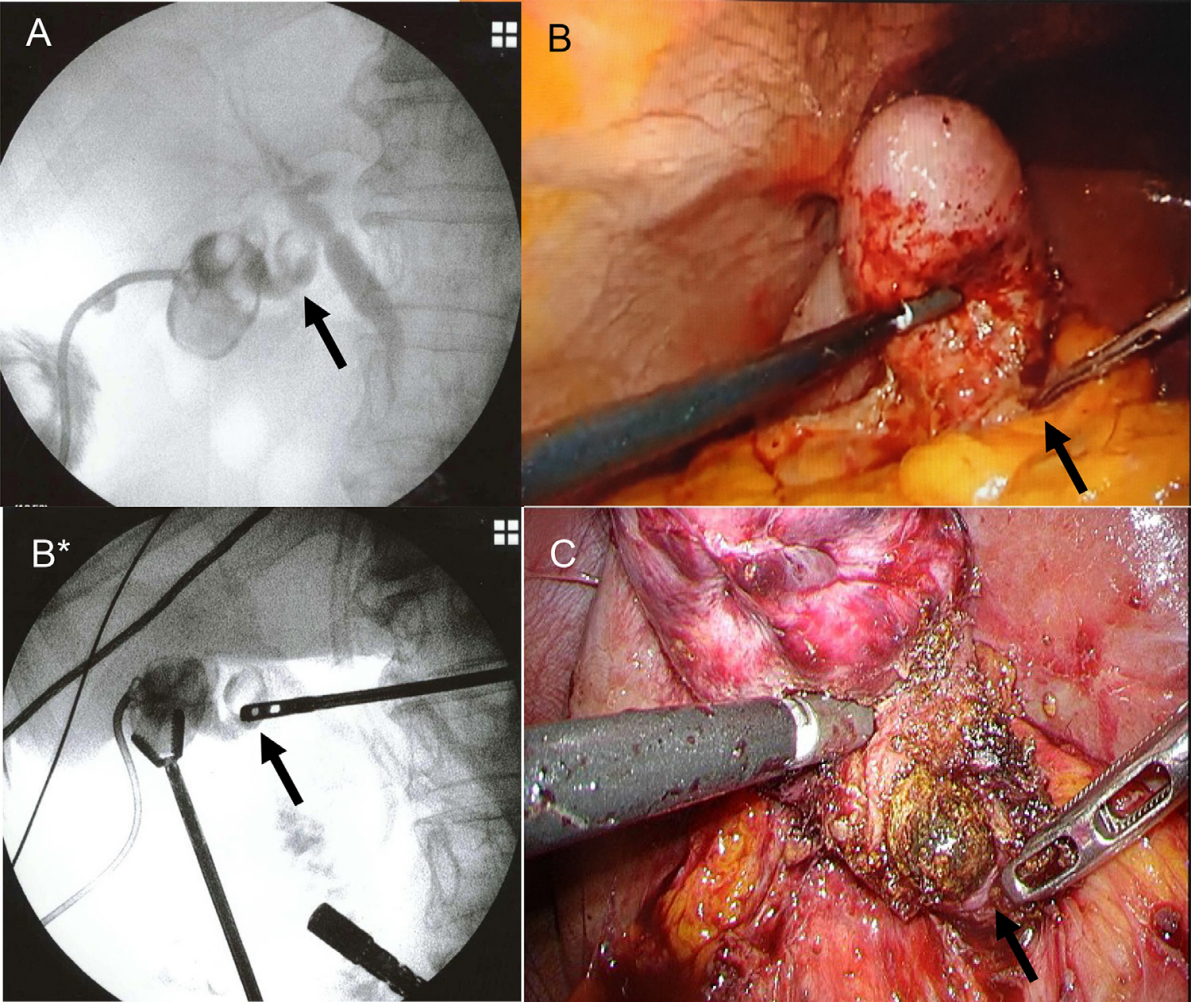
Modified and dynamic IOC to check the laparoscopic image in case 1: (A) Cholangiography via PTGBD tube just before surgery shows biliary anatomy and incarcerated stone (arrow). (B) Laparoscopic image shows the suspected position of the incarcerated stone, which is indicated by the tip of the grasping forceps (arrow). (B*) Modified IOC via PTGBD shows the precise position of the incarcerated stone indicated by the tip of the grasping forceps (arrow). (C) Laparoscopic image shows the incarcerated stone, which is exposed after the incision of the gallbladder wall (arrow). IOC, intraoperative cholangiography; PTGBD, percutaneous transhepatic gallbladder drainage

**Fig. 6:**
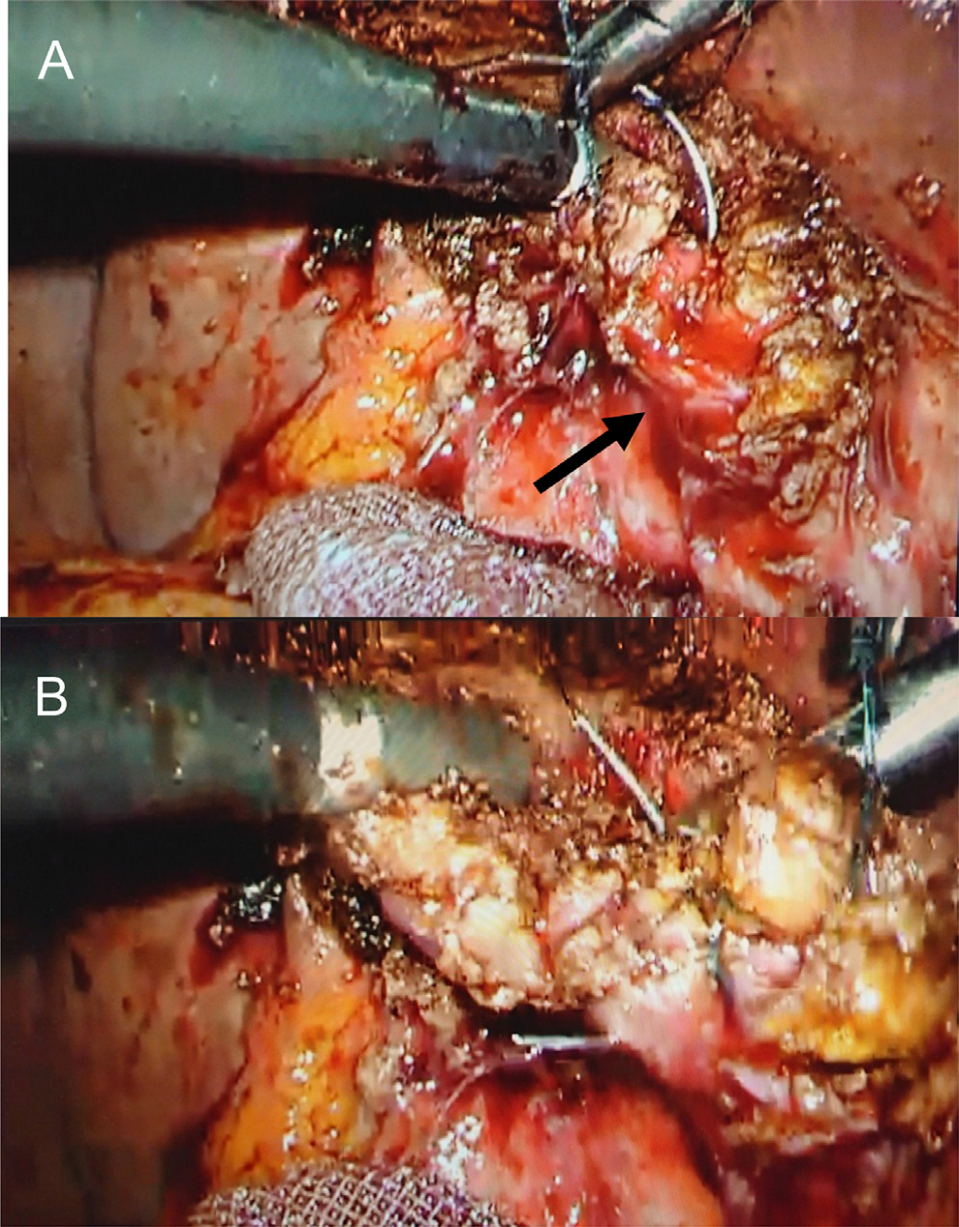
(A) Laparoscopic image shows the stump of the gallbladder infundibulum (arrow). (B) Laparoscopic image shows that the stump is almost sutured and closed with 000 barbed threads.

Under general anesthesia, the fluoroscopic examination was performed using a mobile C-arm image intensifier. Cholangiography via PTGBD tube just before surgery showed biliary anatomy and an incarcerated stone (Fig. 5A). Then a 4-port laparoscopic surgery was performed. Laparoscopy revealed adhesions around the gallbladder and severe inflammatory changes at the Calot's triangle. First, we used a dynamic IOC via the PTGBD tube to move the tip of the grasping forceps to determine the position of the incarcerated stone on the laparoscopic image (Figs. 5B and B*). After that, careful dissection around the incarcerated stone was performed. The segment IV diagonal-line approach [6] could not be performed due to severe fibrosis. Circumferential dissection of the infundibulum was also difficult. For this reason, the LSC policy was adopted. A gallbladder wall incision was made just above the incarcerated stone to extract it (Fig. 5C). Then the body of the gallbladder was resected as much as possible. The PTGBD tube was removed. The cystic artery was not affected. The stump of the gallbladder infundibulum was sutured and closed with 000 barbed threads (Figs. 6A and B [7]). The remaining gallbladder mucosa was cauterized with coagulation using a monopolar electrocautery device. The amount of blood loss was 20 mL, and the operation time was 201 minutes. The patient's postoperative course was uneventful and was discharged 5 days after LSC.

## Case 2

A 57-year-old male with a complaint of epigastric pain was referred to our department requesting laparoscopic cholecystectomy. He had undergone endoscopic sphincterotomy for cholecystocholedocholithiasis and cholangitis 6 months ago. Laboratory tests showed C-reactive protein 0.43 mg/dL (<0.16), which was slightly elevated, but other data were normal. Abdominal CT and MRI revealed multiple stones in the gallbladder and wall thickening. Under general anesthesia, a 4-port laparoscopic surgery was performed. Laparoscopy revealed severe inflammatory changes at the Calot's triangle. The intraoperative diagnosis was severe chronic cholecystitis, so-called difficult gallbladder. Therefore, the LSC policy was adopted.

**Fig. 7:**
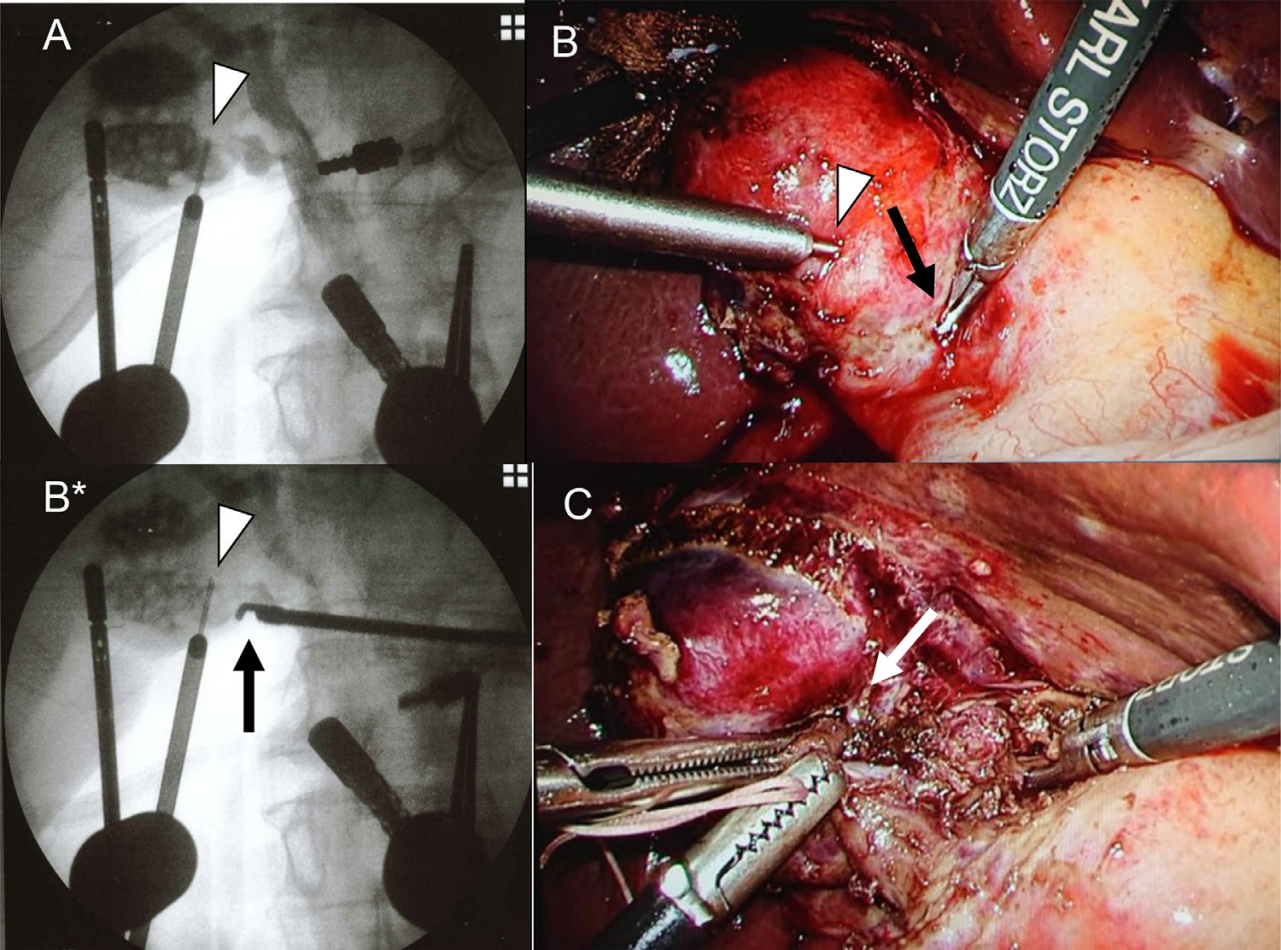
Modified and dynamic IOC to check the laparoscopic image in case 2: (A) Modified IOC by puncturing the gallbladder shows bile duct anatomy and puncture needle (white arrowhead). (B) Laparoscopic image shows the tip of the grasping forceps (arrow). The puncture needle is indicated by a white arrowhead. (B*) Modified IOC via puncture needle (arrowhead) shows the incision line indicated by the tip of the grasping forceps (arrow). It can be confirmed that the incision line is away from the bile duct. (C) Laparoscopic image shows that the dissection around the infundibulum (white arrow) is completed. IOC, intraoperative cholangiography.

First, cholangiography by puncturing the gallbladder body was performed as modified IOC (Fig. 7A). The incision line on the laparoscopic image was determined by moving the tip of the grasping forceps under modified IOC (Figs. 7B and B*). After that, careful dissection around the gallbladder neck was performed (Fig. 7C). The cystic artery could be dissected and separated. Dissection around the gallbladder neck was performed and separated using an automatic stapling system. Then the body of the gallbladder was removed as much as possible. The remaining gallbladder mucosa was cauterized with coagulation using a monopolar electrocautery device. The amount of blood loss was 70 mL, and the operation time was 210 minutes. The patient's postoperative course was uneventful. He was discharged 5 days after LC.

## Discussion

We reported a modified and dynamic IOC procedure via a PTGBD tube or a punctured needle that could identify biliary anatomy, incarcerated stone, and safe incision line during LSC. The modified and dynamic IOC provided direct evidence of the target point of dissection and gallbladder wall incision line. There were 3 important issues in these cases: 1. What kind of therapeutic strategy should be applied to patients with acute cholecystitis complicated with a pericholecystic abscess? 2. Is LSC appropriate for acute cholecystitis undergoing PTGBD? 3. What are the benefits of the modified and dynamic IOC during LSC for difficult gallbladders?

Gallbladder perforation is a potential complication of acute cholecystitis. Increased intraluminal pressure within the gallbladder lumen makes the gallbladder wall ischemic and prone to perforation. Perforation occurs in 2%-11% of acute cholecystitis patients [8,9]. Nonfundal perforations are easily sealed by the omentum, the intestine, or other surrounding tissues. The inflammation is thus confined to the upper right quadrant with the formation of a pericholecystic abscess, however, its treatment is more complicated and time-consuming than common acute cholecystitis. CT or MRI is a noninvasive imaging modality that is useful in evaluating pericholecystic abscess [10,11]. When the pericholecystic abscess is present, the gallbladder lumen communicates with the abscess through the disrupted gallbladder wall. Therefore, PTGBD is effective as an initial treatment for acute cholecystitis with pericholecystic abscess.

The gallbladder after PTGBD, which causes fibrosis around the PTGBD tube, is one of the risk factors for difficult gallbladders [3]. In addition, laparoscopic surgery is more difficult in the case of a pericholecystic abscess, as in case 1, because fibrosis develops around the abscess during the healing process. The rate of bile duct injury during laparoscopic cholecystectomy has not decreased despite advances in endoscopic surgery [5]. Severe inflammation has been reported as one of the causes of bile duct injury [12]. Some surgeons would attempt to perform total cholecystectomy even for difficult gallbladders, however, it is not a minimally invasive surgery. They may unexpectedly face difficulties in detaching the posterior wall of the gallbladder. Adherence to total cholecystectomy can cause injury to the liver bed, increased bleeding, and bile leakage. Aiming for zero bile duct injury, Tokyo guidelines [13] and the Society of American Gastrointestinal and Endoscopic Surgeons guidelines [14,15] recommend LSC as bailout surgery for difficult gallbladders. LSC has been extensively used over recent years, and its effectiveness for complicated cholecystitis has been reported [4,12]. Ie et al. [16] reported that severe fibrosis and adhesion render dissection difficult during laparoscopic surgery for grade II or III acute cholecystitis after PTGBD, and aggressive adoption of LSC increased the completion rate of laparoscopic surgery. Shwaartz et al. [4] reported the usefulness of LSC for difficult gallbladders, however, they rarely performed an IOC. In their series, 1 patient presented with postprocedural mild cholangitis due to cystic stump stones. We believe that confirmation by modified IOC is also useful in preventing residual stones in the cystic duct.

Gallbladder stump preparation is most important in LSC [17]. A convenient incision line should be set to omit unnecessary incisions to reach the desired incarcerated stone, remove the stone, and suture the stump. The purpose of modified and dynamic IOC is to reach the target site safely with minimal effort and in the shortest time. Kuwabara et al. [18] also reported that they often performed cholangiography by puncturing the gallbladder or cannulating the cystic duct from inside the gallbladder after incising the gallbladder neck wall during LSC.

Some authors questioned the usefulness of classic IOC to prevent biliary injury [19]. In the era of laparoscopic cholecystectomy, there is a tendency to leave a long cystic duct stump without IOC. The classic IOC procedure is based on an incision of the cystic duct; misidentification of it leads to bile duct injury. In addition, even in cases with a difficult gallbladder, attempting to dissect the cystic duct can lead to bile duct injury. Classic IOC procedure, which does not lead to bile duct injury avoidance, needs to be modified. We have defined an IOC that does not open the cystic duct as a modified IOC, in contrast to the classic IOC. Modified IOC methods include the PTGBD tube method, the infundibulum puncture method, the infundibulum cannulation method, and IOC using endoscopic nasobiliary drainage tube [5,20]. Modified IOC is applicable not only to mild cases of inflammation but also to severe cases. Dynamically evaluating a modified IOC, which we call modified and dynamic IOC, allows us to see where we are and what we are trying to make an incision. Modified and dynamic IOC can avoid biliary injury. If we aim to zero bile duct injury during laparoscopic surgery; a modified and dynamic IOC confirmation test is mandatory to avoid the bile duct injury as in the case of aircraft accident countermeasures [21]. Therefore, we should not hesitate to perform modified and dynamic IOC as well as preoperative magnetic resonance cholangiopancreatography. We support the strategies for minimizing bile duct injuries for difficult gallbladder by Society of American Gastrointestinal and Endoscopic Surgeons. *“1. Make liberal use of cholangiography or other methods. 2. Consider laparoscopic subtotal cholecystectomy or cholecystostomy tube placement, and/or conversion to an open procedure if conditions around the gallbladder are too dangerous.”* [14,15].

We conclude that the modified and dynamic IOC can identify biliary anatomy, incarcerated stone, and safe incision line during LSC.

## Patient consent

Written informed consent was obtained from each patient for publication of this case report and accompanying images.
